# Supplementary material for: Dissecting task-based fMRI activity using normative modelling: an application to the Emotional Face Matching Task
Source: Commun Biol. 2024 Jul 20;7:888. doi: 10.1038/s42003-024-06573-z (PMC11271583; doi:10.1038/s42003-024-06573-z)
Supplement: Supplementary file 1 — Supplementary Materials [file 42003_2024_6573_MOESM1_ESM.pdf]

SUPPLEMENTARY MATERIALS

Dissecting task-based fMRI activity using normative modelling: an application to the Emotional Face Matching Task

Hannah S. Savage[1,2], Peter C. R. Mulders [1,3], Philip F. P. van Eijndhoven[1,3], Jasper van Oort[1,3], Indira Tendolkar[1,3], Janna N. Vrijsen[1,3,4], Christian F. Beckmann[1,2,5], Andre F. Marquand[1,2]

[1]Donders Institute of Brain, Cognition and Behaviour, Radboud University, Nijmegen, The Netherlands

[2]Department of Cognitive Neuroscience, Radboud University Medical Centre, Nijmegen, The Netherlands

[3]Department of Psychiatry, Radboud University Medical Centre, Nijmegen, The Netherlands

[4]Depression Expertise Centre, Pro Persona Mental Health Care, Nijmegen, The Netherlands

[5]Centre for Functional MRI of the Brain (FMRIB), Nuffield Department of Clinical Neurosciences, Wellcome Centre for Integrative Neuroimaging, University of Oxford, Oxford, UK

Corresponding author:

Hannah S. Savage | Andre F. Marquand

Kappitelsweg 29,

Nijmegen, 6525EN

The Netherlands

E: hannah.savage@donders.ru.nl | andre.marquand@donders.ru.nl

SUPPLEMENTARY MATERIALS

SUPPLEMENTARY METHODS – *Sample Details*:

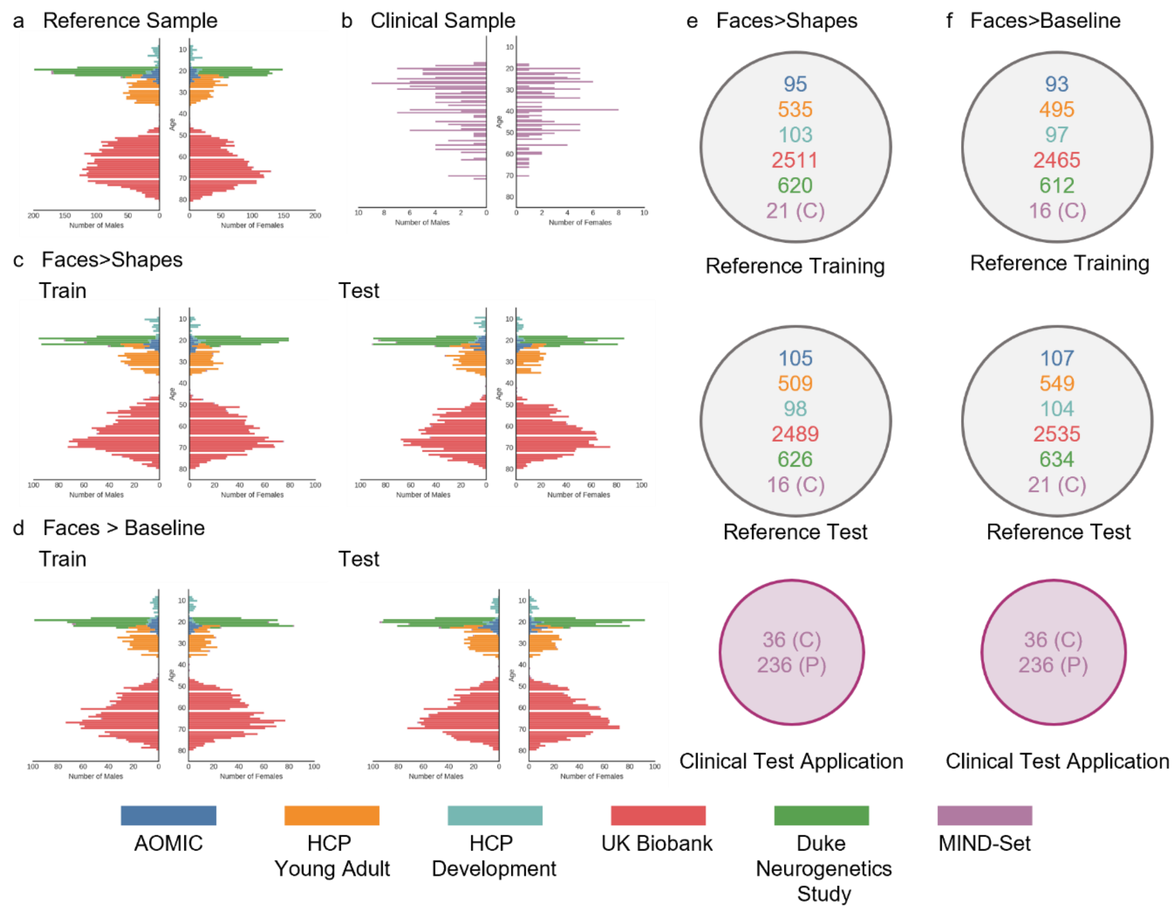

***Supplementary Figure 1**: Age and sex distributions and sample splits.* Age and sex distributions of (a) the total reference sample, (b) the total clinical test sample (MIND-Set) (c) the faces>shapes train (left) and test (right) split, and (d) the faces>baseline train (left) and test (right) split. Details of the number of participants into the Reference model training and test split, and the clinical test application for (c) faces>shapes (e) and faces>baseline (f). C = unaffected MIND-Set control. P = MIND-Set patient.

SUPPLEMENTARY METHODS – *fMRI task paradigms:*

Supplementary Table 1: Additional details of control condition

Site	Control Stimulus	Trials per block/ Blocks/ Total Trials	Trial duration (s)
Human Connectome Project Young Adult	White outline of circles and ovals presented on black background	6/3/18	2
Human Connectome Project Development			
UK Biobank		NA/5/NA	NA
Amsterdam Open MRI Collection Population Imaging of Psychology	Oval stimuli created by pixelating the face stimuli presented on a grey background	6/5/30	when selected or up to 4.8s
Duke Neurogenetics Study	Black filled circles and ovals presented on a white background		4

MIND-Set	Oval stimuli created by pixelating the face stimuli presented on a white background	6/2/12	5

SUPPLEMENTARY METHODS – *Signal coverage of the prefrontal cortex (PFC)*:

Due to air-tissue inhomogeneities which can diminish the acquired BOLD signal to such a degree that no activations are visible, a notorious effect within the ventral PFC, we performed targeted quality control for this extended region. Binary ROI masks were created for the dorsal ventro-medial PFC (d-vmPFC), ventral vmPFC (v-vmPFC), lateral vmPFC (l-vmPFC) and the dorso-medial PFC (dmPFC), as defined by the Harvard Oxford Atlas at 25% probability for atlas regions 25, 27, 33 and 1 respectively (see Supplementary Figure 3B). The percentage of voxels with an absolute value greater than 0 for the contrast faces > shapes within each ROI was determined (i.e. where any signal was present regardless of its relative direction; see Supplementary Figure 1A,B). While most sites had good coverage, the coverage within the ventral and lateral vmPFC regions were particularly variable for the UK Biobank data. We therefore performed this step *only on data from the UK Biobank site*; this selectivity was made possible by the large number of participants we had access to, and our need to include but a fraction of the total available sample. We ranked participants in descending order of the percent of their v-vmPFC, l-vmPFC, d-vmPFC, and dmPFC covered, respectively, and selected the first 5000 participants. We also collected the percentage covered value for a bilateral amygdala ROI mask, but made no exclusion/inclusions on this basis as coverage was very high across all participants and all sites.

Supplementary Table 2: Motion QC

**Site**	**Full sample size**	**Included**	**Excluded**
AOMIC	217	217	0
DNS	1263	1246	17
HCP Young Adult	1044	1044	0
HCP Development	256	256	0
UK Biobank	26167	5000	N/A
MIND-Set	393	389	4

Supplementary Table 3: vmPFC QC

.	Site	Sample size (n)	Mean percentage of ROI covered	Standard deviation
Bilateral Amygdala	AOMIC	217	100	0
	DNS	1246	100	0
	HCP Development	256	99.94936	0.162845
	HCP Young Adult	1044	99.98188	0.098589
	MIND Set	389	99.99966	0.006707
	UK Biobank	26120	99.47363	2.399575
dmPFC	AOMIC	217	99.10345	0.830591
	DNS	1246	99.61285	0.411399
	HCP Development	256	94.6524	2.460686
	HCP Young Adult	1044	96.85686	3.318827
	MIND Set	389	99.069	1.785034
	UK Biobank	26120	99.0493	1.405906
Dorsal vmPFC	AOMIC	217	85.08059	12.23881
	DNS	1246	94.68589	4.406996
	HCP Development	256	72.90154	16.11571
	HCP Young Adult	1044	98.58104	2.192929
	MIND Set	389	99.75218	0.886996
	UK Biobank	26120	94.50698	8.107753
Lateral vmPFC	AOMIC	217	98.90096	1.063159
	DNS	1246	99.82392	0.33135
	HCP Development	256	99.14376	1.003451
	HCP Young Adult	1044	99.46709	0.978404
	MIND Set	389	98.23297	1.745961
	UK Biobank	26120	89.56122	4.919904
Ventral vmPFC	AOMIC	217	96.06479	3.960334
	DNS	1246	99.45099	0.964661
	HCP Development	256	95.999	4.42593
	HCP Young Adult	1044	99.63665	0.85699
	MIND Set	389	98.83508	2.868568
	UK Biobank	26120	61.17452	15.9091

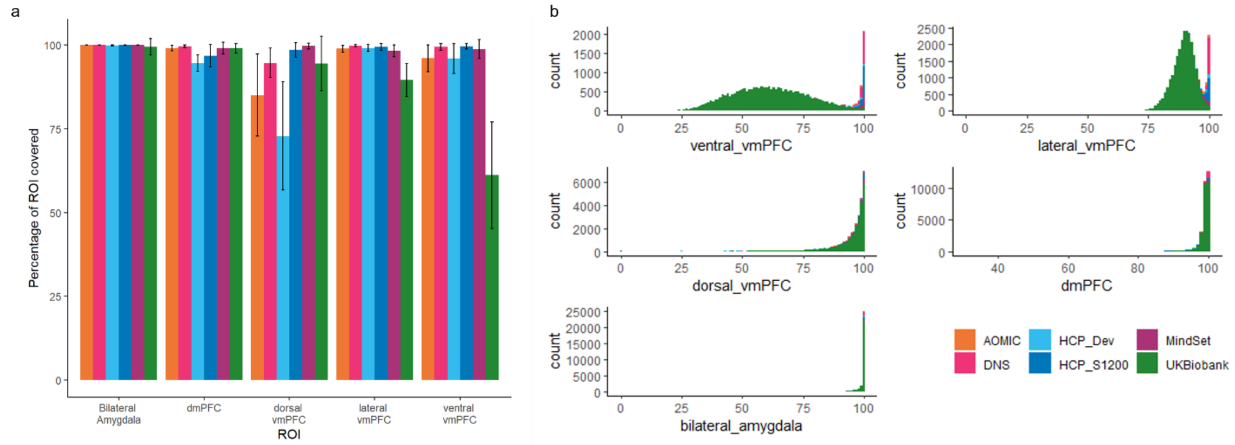

Supplementary Figure 2: vmPFC QC metrics. (a) Mean percentage of each ROI with signal greater than 0, used for quality control. Error bars show +/- standard deviation (b) Stacked histograms (raw participant count) of the percentage of each ROI covered, coloured by site.

SUPPLEMENTARY RESULTS – *Overlap of voxels with low tSNR and the prefrontal cortex QC mask*:

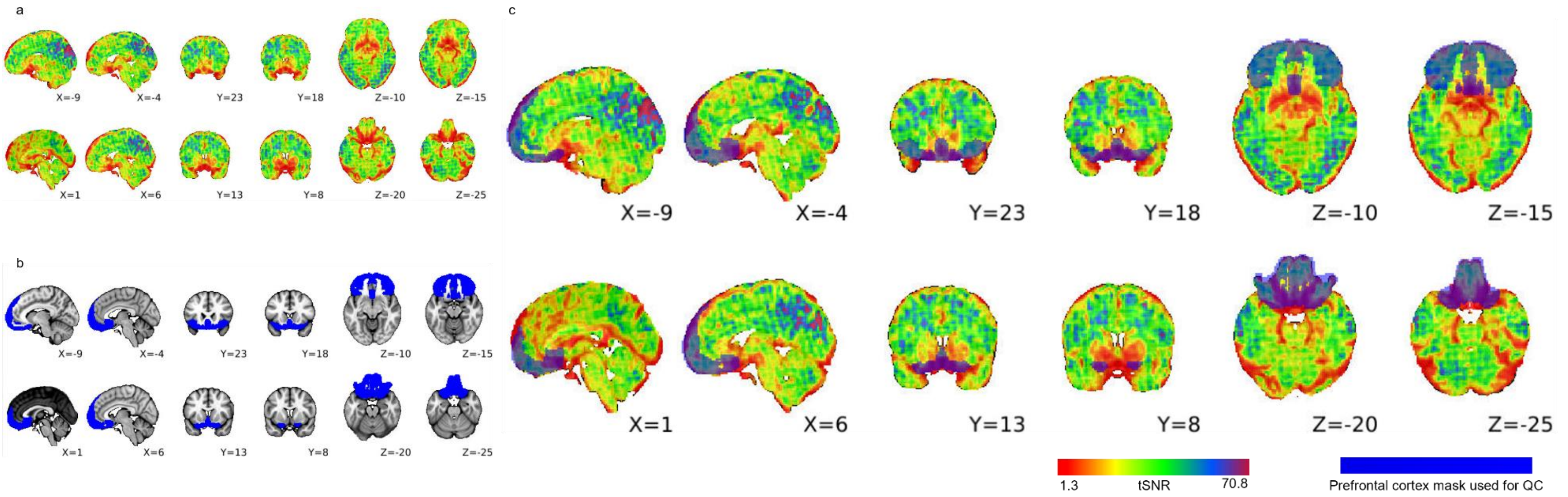

Supplementary Figure 3: The prefrontal cortex mask used for QC overlaps with regions most affected by low tSNR. A representative example UK Biobank participant's tSNR map (a) illustrating regions where tSNR is low. (b) The combined prefrontal cortex mask including the dorsal ventro-medial PFC, ventral vmPFC, lateral vmPFC, and the dorso-medial PFC, as defined by the Harvard Oxford Atlas at 25% probability for atlas regions 25, 27, 33. (c) Overlay of mask on tSNR map.

SUPPLEMENTARY RESULTS – *Task evoked activation*:

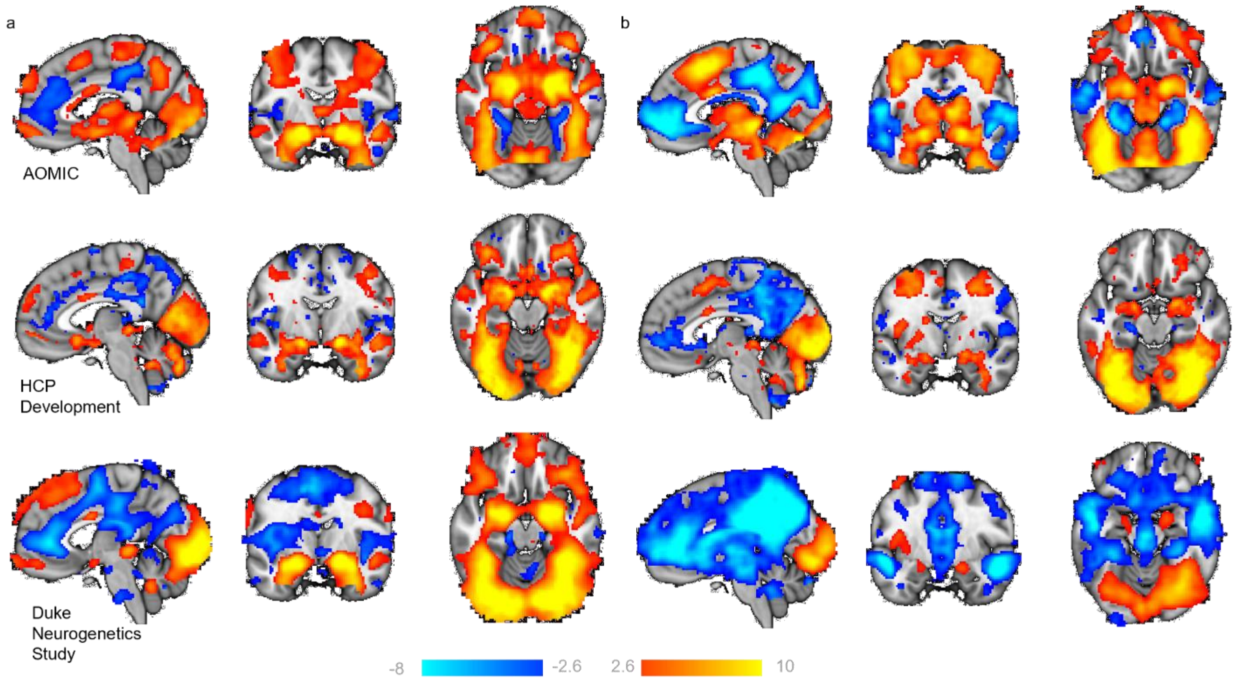

Supplementary Figure 4: Task evoked activation. *R*epresentative groups maps (from Amsterdam Open MRI Collection Population Imaging of Psychology, HCP Development and Duke Neurogenetics Study), illustrating regions where participants show greater BOLD signal (z-statistic maps, thresholded at>±2.6) to (a) faces, as compared to shapes (faces>shapes), and (b) faces, as compared to baseline (faces>baseline). x,y,z = -4,-6,-16.

SUPPLEMENTARY RESULTS – *Evaluation of reference normative models*:

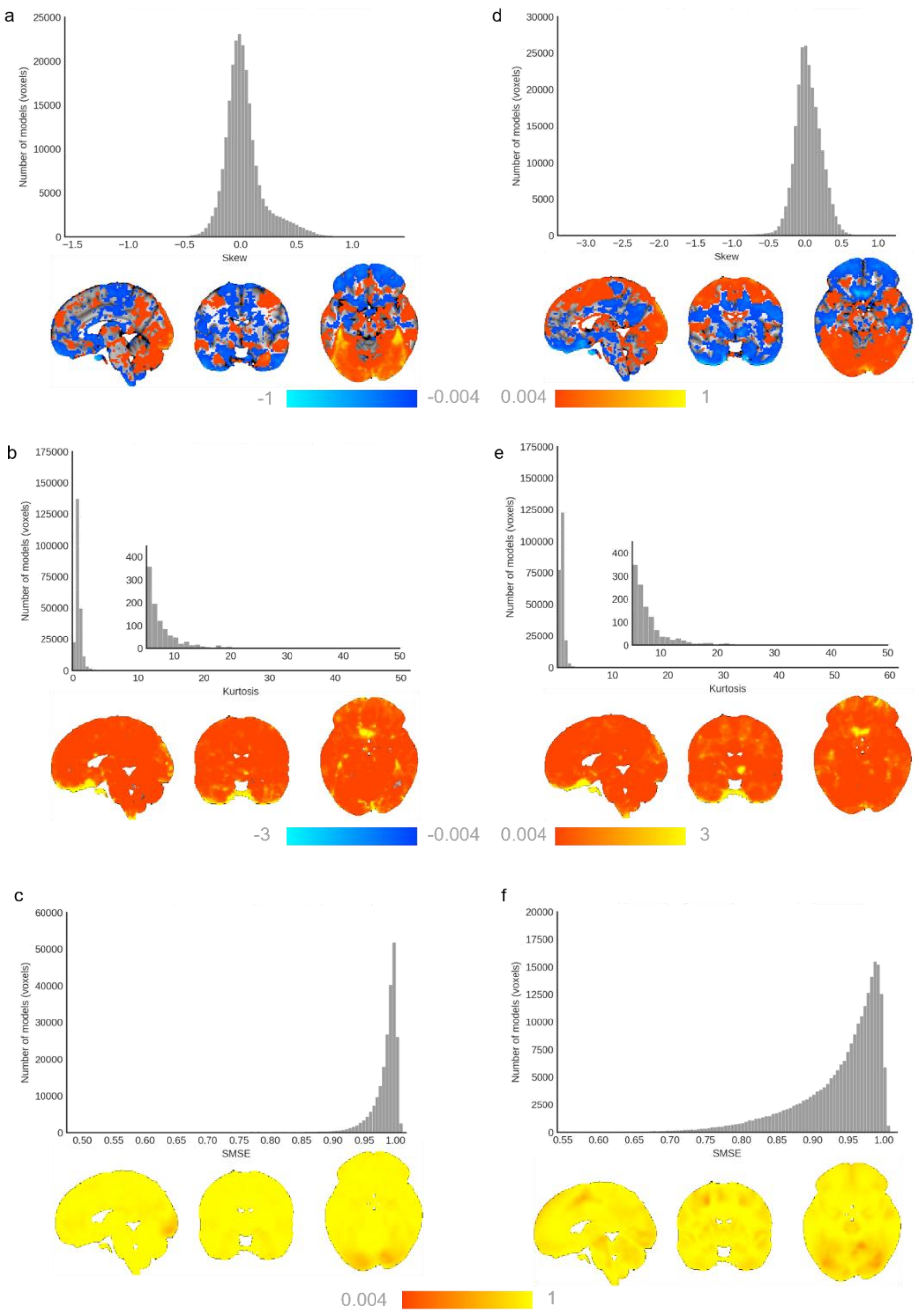

Supplementary Figure 5: Evaluation of the faces>shapes (left) and faces>baseline (right) reference normative models. Histograms show the skew (a,d), kurtosis (b,e), and SMSE (c,f) of the normative models, and their respective illustration on the brain. x,y,z= 4,-6,-16.

SUPPLEMENTARY RESULTS – *Test-Retest analysis for faces>baseline*

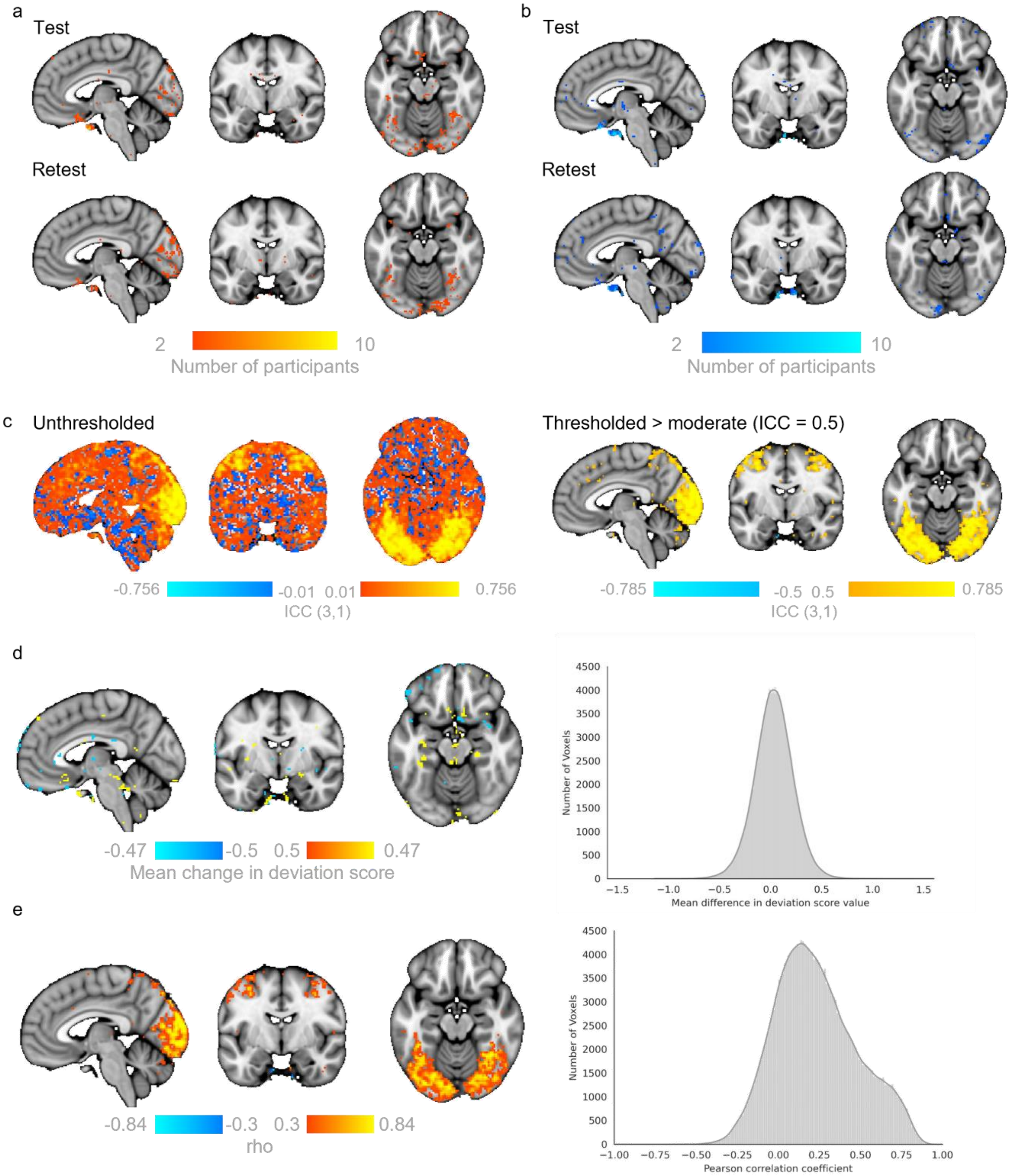

Supplementary Figure 6. Test – Retest reliability of deviation scores for faces>baseline models. Normative Probability Maps illustrate the voxels wherein 2 or more participants had positive (a, hot colours) or negative deviations (b, cool colours) > ±2.6 for the faces > basline normative models in the Test (top rows) and Re-Test (bottom rows) samples. Intra-class correlation coefficients unthresholded (left) and thresholded to show only regions that had a moderate ICC or higher (>0.5; c). Mean within-subject difference per voxel (histogram) illustrated thresholded at >0.5 (i.e. a change greater than half a standard deviation between Test and Retest scans; d). The correlation coefficients (rho) between Test and Retest deviation scores (histogram) illustrated thresholded by the coefficients of determination (rho^2>0.3, e). x,y,z = -4,-6,-16

SUPPLEMENTARY RESULTS – *Additional out of sample test of reference normative models*:

We further validated our reference model with an additional out-of-sample/unseen 5000 participants from UK Biobank. These were the next 5000 participants from the UK Biobank population, as ranked by vmPFC coverage (i.e. decreasing data quality in this region). In most brain regions, the results are very comparable to the initial test sample. This includes good explained variance particularly with visual and extrastriate cortex, and subcortical regions including the amygdala; minimal skew and kurtosis; and frequent large positive deviations in visual and extrastriate cortex regions. Relatedly, we note that the results from the models in the vmPFC region show much greater skew and kurtosis than the original sample, and participants more frequently have large negative deviations in this region. Taken together this most likely reflects the worse signal quality in this region and reinforces the relevant influence of data quality on the deviation scores in this area.

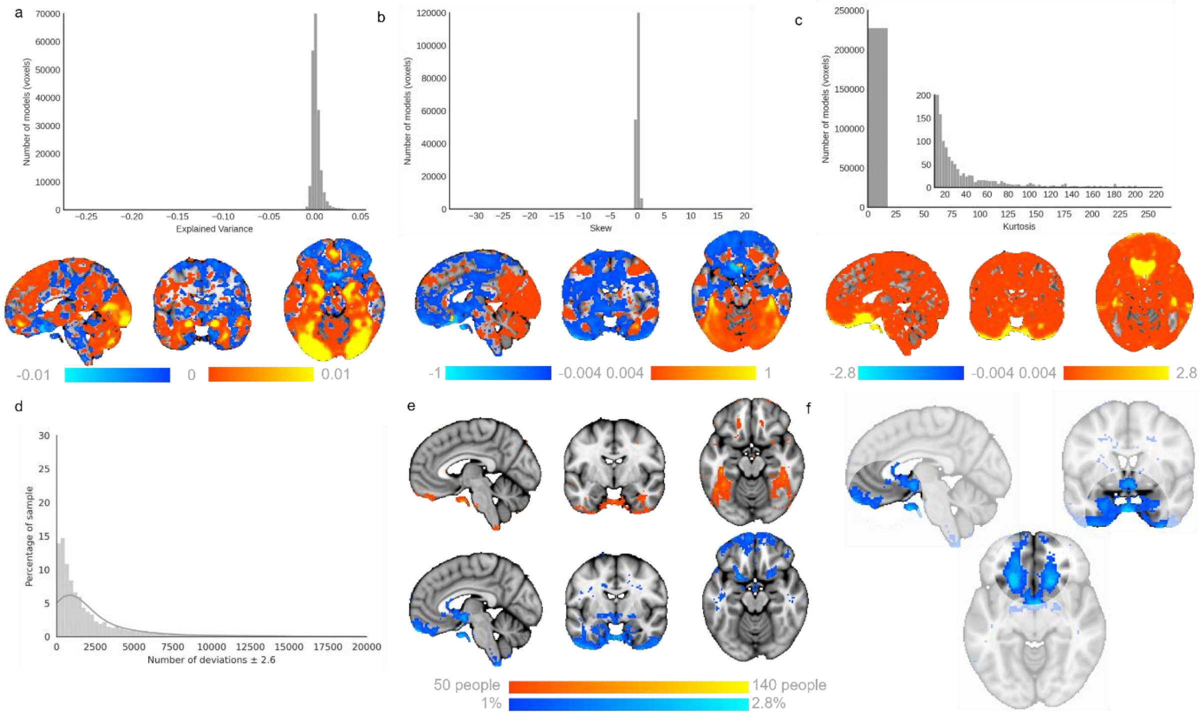

Supplementary Figure 7: Validation of reference normative models in a new sample of 5000 UK Biobank participants. (a) Explained variance, (b) skew, and (c) kurtosis of results when 5000 new UK Biobank participants were tested on the original reference model. (d) Histogram shows the relative frequency of the total number of deviations that a participant has for each model. (e) Normative Probability Maps illustrate the percentage of participants of the total sample who had positive (hot colours) or negative deviations (cool colours) > ±2.6 within each voxel, for the faces > shapes, where (f) highlights the frequent negative deviations within the vmPFC/OFC region. x,y,z = -4,-6,-16

SUPPLEMENTARY RESULTS – *Evaluation of normative models when applied to MIND-Set cohort*:

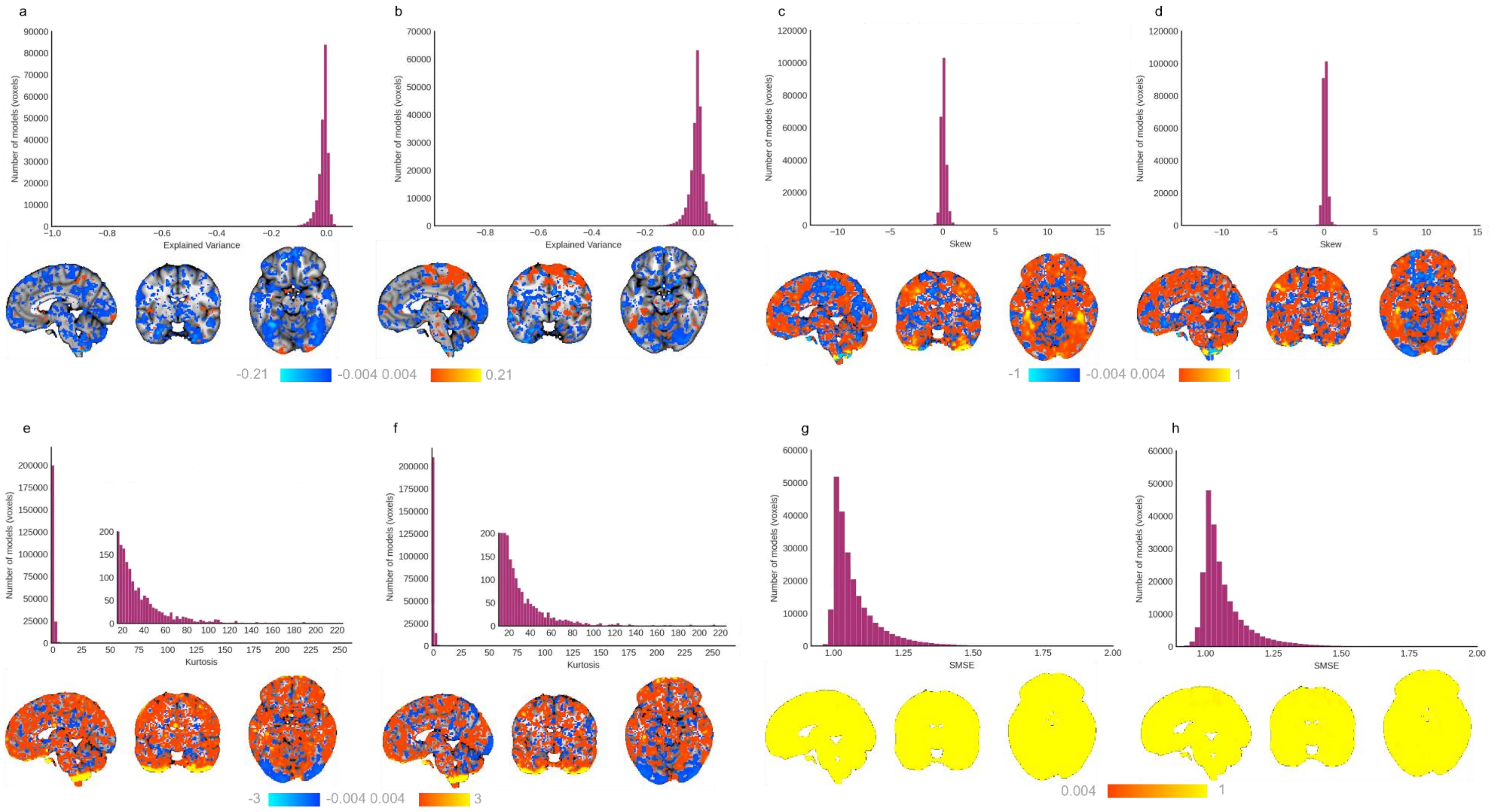

Supplementary Figure 8: Evaluation of the faces>shapes (left) and faces>baseline normative models when applied to MIND-Set cohort. Histograms show the explained variance (a,b), skew (c,d), kurtosis (e,f), and SMSE (g,h) of the clinical data, as tested on reference normative models of EFMT related BOLD activation, and their respective illustration on the brain. x,y,z= 4,-6,-16.

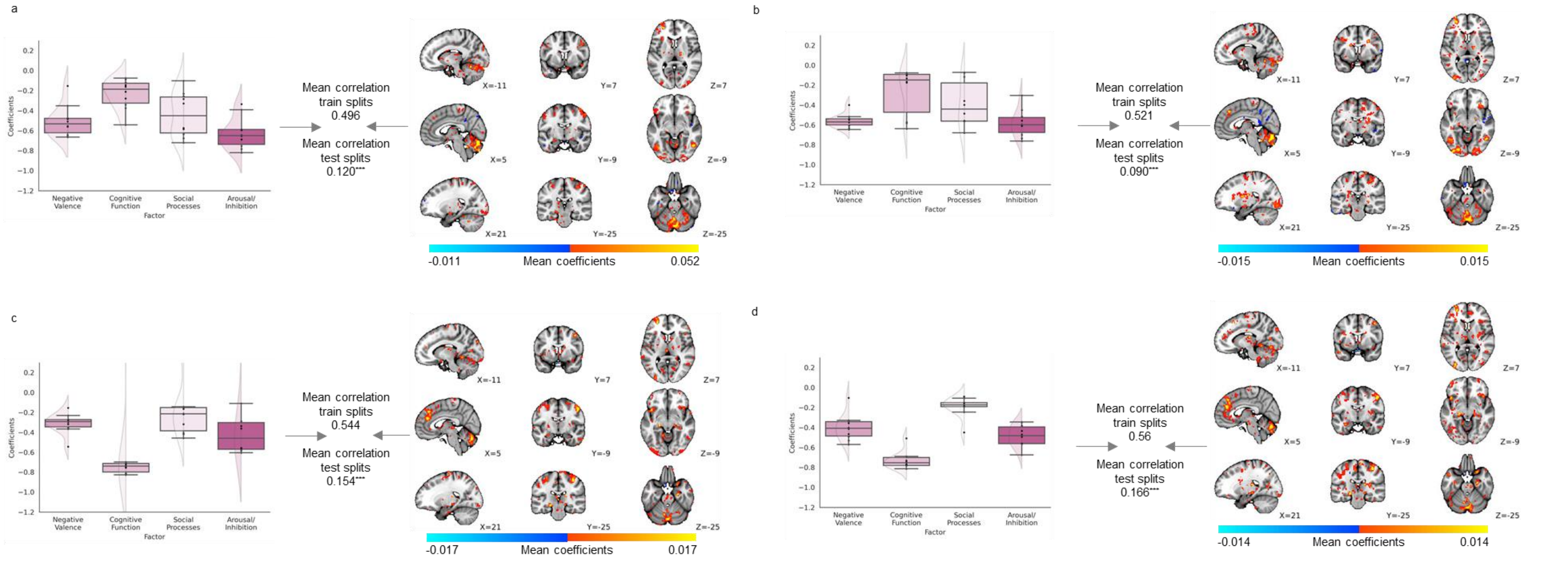

Supplementary Figure 9: Sparse canonical correlation analyses (SCCA) between functional domains, and deviation scores from faces>shapes or faces>baseline normative models constrained to grey matter (left) or whole-brain (right). Weights per factor to latent variable of psycho-social functioning, canonical correlation between 4 functional domains and deviation scores, and their relative mean voxel-wise weights to latent variable of deviation scores from (a,b) faces>shapes, and (c,d) faces>baseline normative models (regularisation 10%). Box plot whiskers (error bars) show 1.5 times the interquartile range from the lower or upper quartile. All results are statistically significant with 1000-fold permutation tests (*** = *p*<0.001).

SUPPLEMENTARY RESULTS – *Location of deviations for diagnoses:*

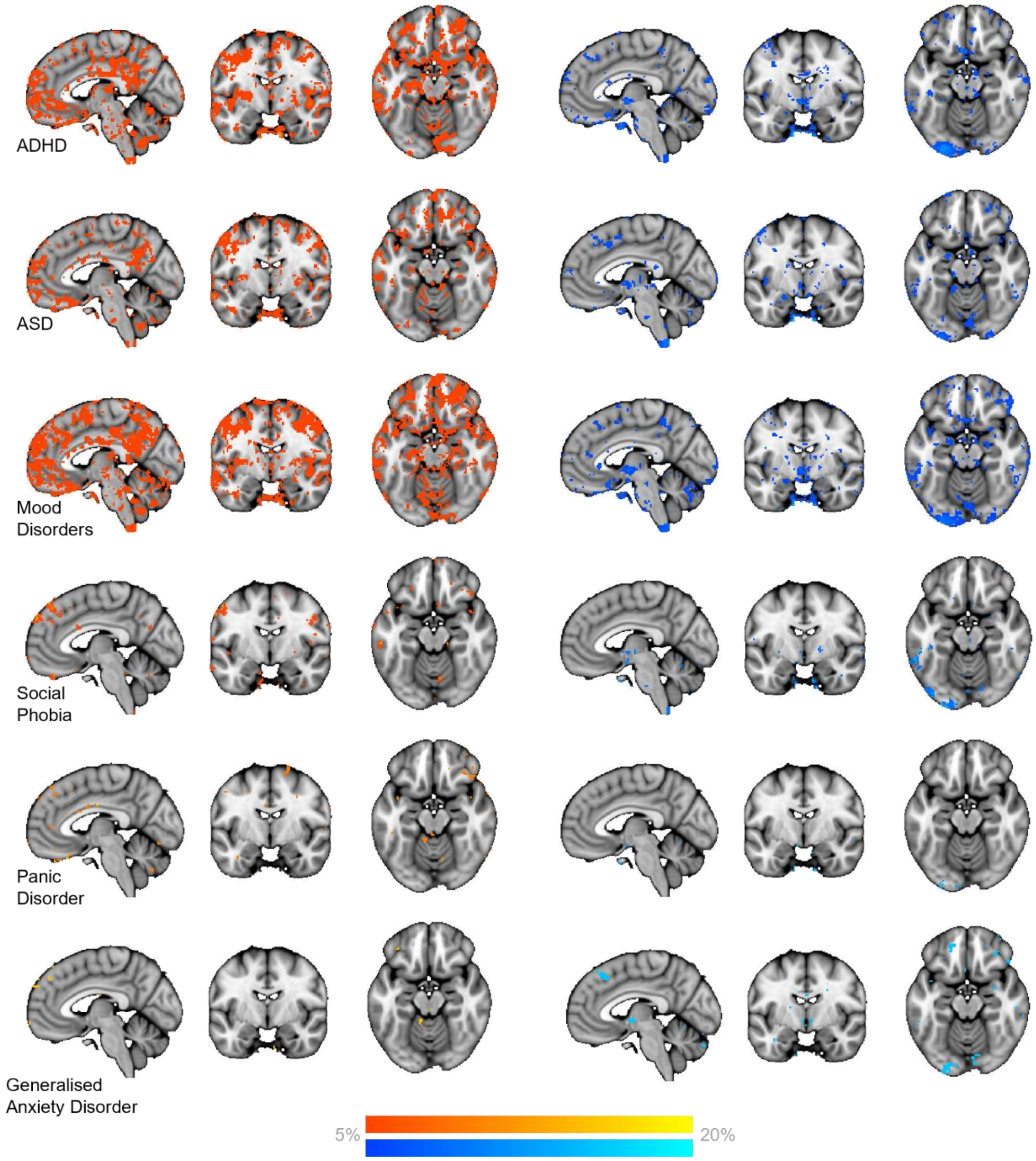

Supplementary Figure 10: Heterogeneous location of deviations in predicted BOLD signal for different types of neurodivergence, and mental health diagnoses. Maps illustrate the percentage of participants with a neurodivergence or mental health condition who had positive (left; hot colours) or negative deviations (right; cool colours) > ±2.6 within each voxel [minimum = %5 of sample, or 1 participant where 5% was a participant count less than 1, maximum = 20% of disorder sample size]. x,y,z, = -4,-6,-16.

SUPPLEMENTARY RESULTS – *Location of deviations for increasing levels of co-occurring diagnoses:*

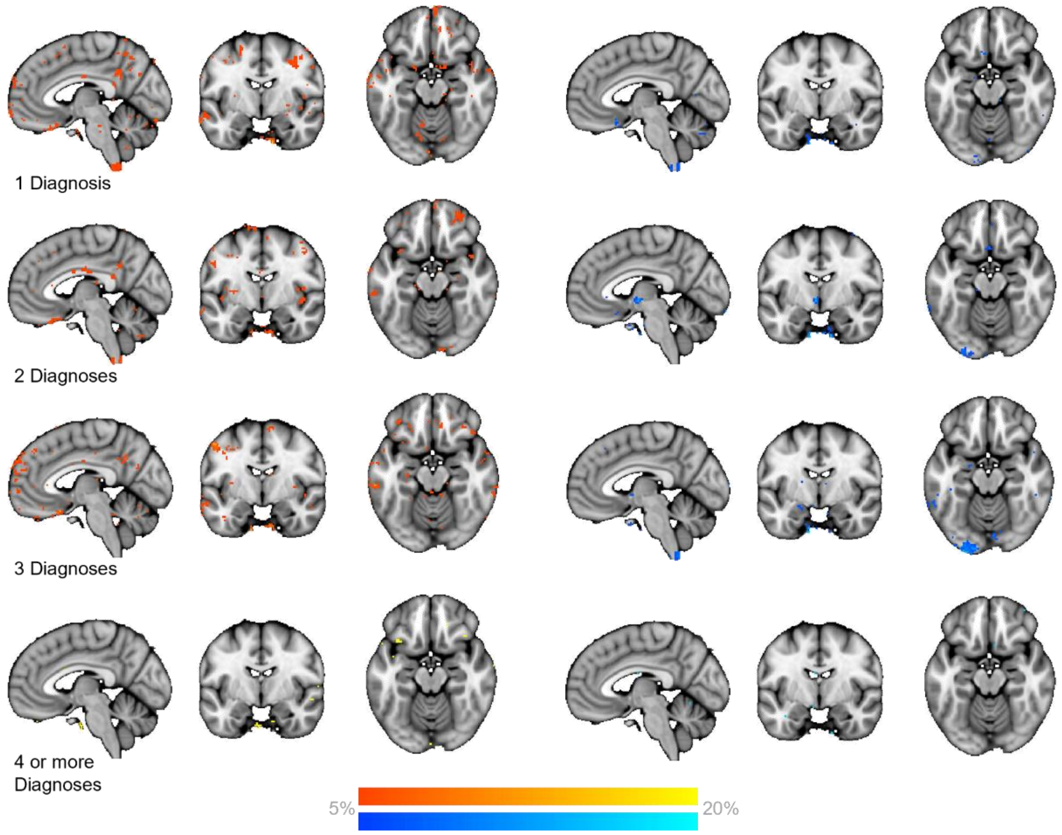

Supplementary Figure 11: Heterogeneous location of deviations in predicted BOLD signal for increasing levels of co-occurring diagnoses. Maps illustrate the percentage of participants with a neurodivergence or mental health condition who had positive (left; hot colours) or negative deviations (right; cool colours) > ±2.6 within each voxel [minimum = %5 of sample, or 1 participant where 5% was a participant count less than 1, maximum = 20% of sample size]. x,y,z = -4,-6,-16
